# *Candidozyma auris* susceptibility from Michigan hospitals to manogepix, rezafungin and nine other antifungal agents

**DOI:** 10.1093/jacamr/dlag089

**Published:** 2026-05-27

**Authors:** Katherine S Klamer, Ashish Bhargava, Leonard Johnson, Mamta Sharma, Louis D Saravolatz

**Affiliations:** Thomas Mackey Center for Infectious Disease Research, Henry Ford St.John Hospital, Grosse Pointe Woods, MI, USA; Thomas Mackey Center for Infectious Disease Research, Henry Ford St.John Hospital, Grosse Pointe Woods, MI, USA; Collage of Medicine, Central Michigan University College of Medicine, Mt. Pleasant, MI, USA; School of Medicine, Wayne State University School of Medicine, Detroit, MI, USA; Thomas Mackey Center for Infectious Disease Research, Henry Ford St.John Hospital, Grosse Pointe Woods, MI, USA; Collage of Medicine, Central Michigan University College of Medicine, Mt. Pleasant, MI, USA; School of Medicine, Wayne State University School of Medicine, Detroit, MI, USA; Thomas Mackey Center for Infectious Disease Research, Henry Ford St.John Hospital, Grosse Pointe Woods, MI, USA; Collage of Medicine, Central Michigan University College of Medicine, Mt. Pleasant, MI, USA; School of Medicine, Wayne State University School of Medicine, Detroit, MI, USA; Thomas Mackey Center for Infectious Disease Research, Henry Ford St.John Hospital, Grosse Pointe Woods, MI, USA; Collage of Medicine, Central Michigan University College of Medicine, Mt. Pleasant, MI, USA; School of Medicine, Wayne State University School of Medicine, Detroit, MI, USA

## Abstract

**Background:**

*Candidozyma auris* (*C. auris*) is a resistant fungal pathogen with high mortality.

**Objectives:**

To evaluate the *in vitro* activity of 11 antifungal agents against *C. auris* from multiple hospitals.

**Methods:**

MIC was determined by microdilution testing for amphotericin B, anidulafungin, caspofungin, manogepix, micafungin, rezafungin and five triazoles. Sanger sequencing was performed on one echinocandin resistant isolate.

**Results:**

There were 84 *C. auris* isolates from Michigan that were compared with 27 organisms from external sources. Manogepix had the lowest MIC_90_ (0.03 mg/L). The next most active agent was micafungin. The echinocandins demonstrated a 98.8% susceptibility. A single isolate was found to have a S639F mutation. Amphotericin B demonstrated a 97.6% susceptibility. The MIC_90_ demonstrated values of ≤0.5 mg/L for isavuconazole, itraconazole and posaconazole. When separating by colonization versus infection isolates, the MIC_90_ values were identical for all agents, except micafungin, which showed 0.25 versus 0.12 mg/L, respectively. When comparing the reference isolates with Michigan isolates, the MIC_90_ was identical for echinocandins and manogepix. Differences noted were with amphotericin B, for which 85.2% were susceptible for reference isolates compared to 97.6% from Michigan isolates, and fluconazole, where 25.9% were susceptible for the reference isolates compared with 4.8% from Michigan isolates.

**Conclusions:**

Manogepix and echinocandins demonstrated the greatest activity against *C. auris* irrespective of hospital and were like the reference isolates. Amphotericin B demonstrated greater activity compared with published studies. Fluconazole has poor activity; the activity of other triazoles remains unclear.

## Introduction

*Candidozyma auris* (*C. auris*), formerly known as *Candida auris,* is a multidrug resistant fungal pathogen.^1^ Although only identified in 2009, it has emerged as a worldwide threat to patients in healthcare facilities.^2,3^ This pathogen has become widespread in the USA with six genetically distinct clades identified at this time. In Michigan, clades I, II, III and IV have been identified with clade IV being the most prevalent when evaluating 435 isolates of *C. auris.*^4^ In addition, virulence and antifungal susceptibility may vary among clades.^5^ In view of the severity of illness and mortality associated with *C. auris* infections, optimal therapy needs additional guidance in terms of *in vitro* data and identification of new antifungal agents.^6^ Furthermore, regional variation of susceptibility of *C. auris* has not been extensively studied. Our study evaluated *in vitro* activity of 11 antifungal agents representing multiple classes against isolates colonizing or infecting hospitalized patients in seven southeastern Michigan hospitals and compared them to reference isolates from sites outside of Michigan. This study also included two recently developed antifungal agents, manogepix and rezafugin, in determining their activity against *C. auris.*

## Materials and methods

### Organisms

A total of 111 isolates of *C. auris* were studied that included 84 non-duplicate *C. auris* isolates from seven hospitals in southeastern Michigan. Sources of the isolates are screens (axilla/groin), urine, blood, bone, wounds, tissue and bile fluid. Among the clinical isolates, there was information related to treatment and outcome on six patients with positive blood cultures. Three of these patients had clearance of their *C. auris* with anidulafungin at 1, 6 and 9 days of treatment. The other three received no treatment and died between days 1 and 3 of hospitalization before *C auris* was confirmed from their blood cultures. Comparator organisms were collected from the Biological and Emerging Infections Research Resources Program (BEI) (*n* = 4), ATCC (*n* = 4) and the CDC/FDA Antimicrobial Resistance Isolate Bank (*n* = 19).

### Isolation and storage

*C. auris* isolates are initially processed on sabouraud dextrose agar at 35°C and single colony identity was confirmed using CHROMagar^™^ (CHROMagar^™^, Paris France) and MALDI-TOF-MS (Bruker Daltonics, Billerica, MA, USA) or by CHROMagar^™^ and Vietk-2 yeast identification card (bioMérieux, Marcy-l'Étoile, France). The isolates were stored at −80°C in tryptic soy broth with 20% sterile glycerol.

### Antifungal susceptibility testing

The antifungals tested were amphotericin B, anidulafungin, caspofungin, fluconazole, isavuconazole, itraconazole, manogepix, micafungin, posaconazole, rezafungin and voriconazole. Broth microdilution antifungal susceptibility testing was performed in accordance with the CLSI M27 Reference Method for Broth Dilution Antifungal Susceptibility Testing of Yeasts.^7^ Manogepix was received from Basilea Pharmaceutica (Allschwil, Switzerland). Thermoscientific Sensititre Yeast One Antimicrobial Susceptibility Testing YO11 Plates (Thermo Fisher Scientific, Waltham, MA, USA) were used for testing amphotericin B, anidulafungin, caspofungin, fluconazole, isavuconazole, itraconazole, micafungin, posaconazole, rezafungin and voriconazole. Manogepix was prepared separately from the YO11 plates. Sterile RPMI 1640 broth was used for both, however, in the panel testing the broth was prepared by the manufacturer of the panels (Thermo Fisher Scientific, Waltham, MA, USA). For both YO11 plates and manogepix panels, inoculum was prepared at a 0.5 McFarland standard read at a 530-nm wavelength. The plates were incubated at 35°C for 24 hours. Final reading of all plates was carried out using a convex mirror plate reader.

At this time, many breakpoints have not been established for *C. auris.* However, rezafungin is the only CLSI approved breakpoint that is considered susceptible at ≤0.5 mg/L.^8^ The CDC defined tentative breakpoint guidelines on which we based our percentage susceptibility. Fluconazole has a recommended breakpoint of ≥32 mg/L. For itraconazole and second generation triazoles, the CDC and CLSI have no defined breakpoints. Amphotericin B has a recommended breakpoint of ≥2 mg/L. Anidulafungin and micafungin have a tentative breakpoint of ≥4 mg/L.^9^

### FKS1 gene analysis

DNA extraction was performed using the Thermo Scientific Yeast DNA Extraction Kit (Thermo Scientific, Rockford, IL, USA). Primer sequences for hot spot 1 (HS1) was GCCATCTCGAAGTCTGCTCA (forward sequence) and TGACAATGGCATTCCACACCT (reverse sequence), which came from accession number XM_018312389.1.^10^ For the amplification of FKS1, the cycling included initial denaturation at 95°C for 5 min, 45 cycles of 95°C for 10 s, annealing at 60°C for 30 s and extension at 72°C for 1 min followed by a 72°C 5 min final extension.^10^ PCR purification and Sanger sequencing was performed at Eurofins Genomics (Louisville, KY, USA). Benchling was used to align the DNA sequence to the reference locus B9J08_000964 (*Candida* Genome Database).^11^

## Results

### Antifungal susceptibility testing

The *in vitro* activity of the 11 tested antifungal agents against all *C. auris* isolates tested (*n* = 84) is provided in Table 1. Manogepix had the lowest MIC_90_ (0.03 mg/L) compared with the other 10 agents. The next most active agent was micafungin. All the echinocandins demonstrated a 98.8% susceptibility. Notably, amphotericin B demonstrated a 97.6% susceptibility. There are no EUCAST or CLSI breakpoints for triazoles except for fluconazole at this time and thus the percentage susceptibility could not be reported. However, the MIC_90_ demonstrated values of ≤0.5 mg/L for isavuconazole, itraconazole and posconazole. When separating isolates by source of recovery, colonization versus infections (Tables 2 and 3) MIC_90_ were identical except for micafungin that showed 0.25 versus 0.12 mg/L, respectively, for colonizing versus infection isolates, which is within the margin of error for antifungal testing.

**Table 1.** Southeastern Michigan hospital *C. auris* isolates (*n* = 84)

Antifungal	MIC_50_ (mg/L)	MIC_90_ (mg/L)	Percentage susceptible (%)	Range
Amphotericin B	1	1	97.6	0.5–2
Fluconazole	64	64	4.8	8–>64
Isavuconazole	0.5	0.5	N/A^a^	0.015–1
Itraconazole	0.25	0.5	N/A^a^	<0.015–0.5
Posaconazole	0.12	0.25	N/A^a^	<0.008–0.5
Voriconazole	0.5	2	N/A^a^	0.06–>2
Anidulafungin	0.25	0.25	98.8	0.03–4
Caspofungin	0.25	0.25	98.8	0.06–2
Micafungin	0.12	0.12	98.8	0.06–>8
Rezafungin	0.12	0.25	98.8	0.015–2
Manogepix	0.015	0.03	N/A^a^	0.004–0.03

^a^Not available (N/A) due to the lack of established breakpoints.

**Table 2.** Southeastern Michigan hospital *C. auris* colonization isolates (*n* = 58)

Antifungal	MIC_50_ (mg/L)	MIC_90_ (mg/L)	Percentage susceptible (%)	Range
Amphotericin B	1	1	96.6	0.5–2
Fluconazole	64	> 64	6.9	8–>64
Isavuconazole	0.5	0.5	N/A^a^	0.015–1
Itraconazole	0.25	0.5	N/A^a^	<0.015–0.5
Posaconazole	0.12	0.25	N/A^a^	<0.008–0.25
Voriconazole	0.5	2	N/A^a^	0.06–>2
Anidulafungin	0.12	0.25	98.3	0.03–4
Caspofungin	0.25	0.25	98.3	0.06–2
Micafungin	0.12	0.25	98.3	0.06–>8
Rezafungin	0.12	0.25	98.3	0.015–2
Manogepix	0.015	0.03	N/A^a^	0.004–0.03

^a^Not available (N/A) due to the lack of established breakpoints.

**Table 3.** Southeastern Michigan hospital *C. auris* infectious isolates (*n* = 26)

Antifungal	MIC_50_ (mg/L)	MIC_90_ (mg/L)	Percentage susceptible (%)	Range
Amphotericin B	1	1	100	0.5–1
Fluconazole	64	> 64	0	64–>64
Isavuconazole	0.5	0.5	N/A^a^	0.06–1
Itraconazole	0.25	0.5	N/A^a^	0.12–0.5
Posaconazole	0.12	0.25	N/A^a^	0.03–0.5
Voriconazole	0.5	2	N/A^a^	0.25–>2
Anidulafungin	0.25	0.25	100	0.12–0.5
Caspofungin	0.25	0.25	100	0.12–0.5
Micafungin	0.12	0.12	100	0.06–0.25
Rezafungin	0.12	0.25	100	0.12–0.25
Manogepix	0.015	0.03	N/A^a^	0.008–0.03

There were 27 reference strains that were used for comparison to demonstrate the reproducibility of our results as well as to provide a comparative reference for our strains versus isolates from other geographic areas (Table 4). The MIC_90_ was identical for anidulafungin, caspofungin, micafungin, rezafungin and manogepix. Differences noted were with amphotericin B for which 85.2% were susceptible for reference isolates compared with 97.6% from Michigan isolates and fluconazole where 25.9% for the reference isolates were susceptible compared with 4.8% from Michigan isolates. The triazoles MIC_90_ was identical or a 2-fold dilution difference when comparing the isolates from Tables 1 and 4.

^a^Not available (N/A) due to the lack of established breakpoints.

**Table 4.** *C. auris* reference isolates (*n* = 27)

Antifungal	MIC_50_ (mg/L)	MIC_90_ (mg/L)	Percentage susceptible (%)	Range
Amphotericin B	1	2	85.2	0.5–2
Fluconazole	>64	>64	25.9	8–>64
Isavuconazole	0.25	0.5	N/A^a^	0.015–1
Itraconazole	0.25	0.25	N/A^a^	<0.015–0.5
Posaconazole	0.12	0.25	N/A^a^	<0.008–0.25
Voriconazole	1	>2	N/A^a^	0.06–>2
Anidulafungin	0.12	0.25	100	0.03–4
Caspofungin	0.25	0.25	100	0.06–2
Micafungin	0.12	0.12	100	0.06–>8
Rezafungin	0.12	0.25	100	0.015–2
Manogepix	0.03	0.03	N/A^a^	0.004–0.03

^a^Not available (N/A) due to the lack of established breakpoints.

### FKS1 *gene analysis*

The isolate R171 showed reduced echinocandin susceptibility and was evaluated for a *FKS1* mutation. Minimum inhibitory concentrations for R171 are as follows, anidulafungin 4 mg/L (resistant breakpoint is ≥4 mg/L), caspofungin 2 mg/L (resistant breakpoint ≥2 mg/L), micafungin is >8 mg/L (resistant breakpoint ≥4 mg/L) and rezafungin is 2 mg/L (susceptible breakpoint ≤0.5 mg/L). Results from the sequencing showed that the isolate has a serine to phenylalanine amino acid substitution (S639F).

## Discussion

Manogepix demonstrated the greatest *in vitro* activity against isolates for southeastern Michigan as well as reference isolates. However, considering the lack of a defined breakpoint for manogepix this could overstate its activity. Regardless, this is still promising as new antifungals are clearly needed for the treatment of *C. auris* infections. Manogepix is a novel antifungal agent with a unique mechanism of action. Fosmanogepix is a prodrug that is converted *in vivo* to the active drug manogepix. This drug works by inhibiting a crucial fungal enzyme Gwt1, which is necessary for fungal cell wall and cell membrane biosynthesis, making it the first in its class to have this mechanism of action.^10^

The *C. auris* isolates from southeastern Michigan (SE) hospitals also demonstrated greater than 90% susceptibility to amphotericin B, very close to what reference isolates showed in our study (85.2%). However, this is greater than previously published *in vitro* studies, which showed a 70% susceptibility.^5^

Resistance to fluconazole was seen among all isolates from clinical infections. However, other triazoles demonstrated activity of unclear significance as breakpoints are not established from CLSI and EUCAST at this time. Once other breakpoints are established on the other triazoles re-evaluation of that activity will need to be carried out.

Rezafungin and other echinocandins demonstrated that resistance was uncommon and seen in only one isolate. The mutation identified in the S639F substitution associated with echinocandin resistance may be important for clinical therapeutic decisions if this mutation becomes more common in future surveillance studies. Although rezafungin is the newest of echinocandins, it did not demonstrate superior *in vitro* activity against the other available antifungal agents. However, rezafungin still offers an advantage over other echinocandins due to a longer half-life and allows for a dosing of once a week through intravenous administration. This high initial dose can help slow the advance of echinocandin resistance in *C. auris*.

As expected, isolates obtained from screening for colonization demonstrated similar susceptibility as those isolates from documented infections.

Overall, antifungal *in vitro* activity against our southeastern Michigan *C. auris* was similar to *in vitro* activity of antifungal agents against organisms compared from several reference sources except for amphotericin B where Michigan isolates were more susceptible to amphotericin B and less susceptible to fluconazole.

Final conclusions about the clinical activity of antifungal agents against *C. auris* will have to await clinical trials that may take some time to complete. However, regional *in vitro* activity should be performed to determine the variability of antifungal agents against *C. auris* and to guide therapy. Last, new agents such as manogepix need to be evaluated because they may provide alternative treatment options for the treatment of this challenging pathogen.

### Conclusion

Manogepix and echinocandins demonstrated the greatest *in vitro* activity against *C. auris* isolates from both colonizing and infection sites irrespective of hospital of origin and were similar to reference isolates,

Amphotericin B demonstrated greater activity in this study compared with published studies and was similar to reference isolates. Fluconazole has poor activity, however, the activity of other triazoles remains unclear. Further studies comparing larger numbers of isolates from different clades as well as treatment trials should be performed.
